# The acute mesenteric ischaemia (AMESI) study: a matter of incidence

**DOI:** 10.1186/s13054-024-04850-1

**Published:** 2024-03-04

**Authors:** Lorenzo Garzelli, Mathieu Nacher

**Affiliations:** 1 Department of Medical Imaging, Cayenne Hospital Center, University of French Guiana, Avenue des Flamboyants, 97306 Cayenne, French Guiana France; 2 University of French Guiana, Cayenne, France; 3 Clinical Investigation Center Antilles French Guiana (CIC INSERM 1424), Cayenne Hospital Center, Cayenne, French Guiana France

## To the editor,

We read with great interest the recent study by Dr. Reintam Blaser et al. “Incidence, diagnosis, management and outcome of acute mesenteric ischaemia: a prospective, multicenter observational study (AMESI Study)” [1] in which they prospectively identified the occurrence rate of acute mesenteric ischaemia (AMI) among adult patients hospitalized in acute care hospitals in different parts of the world from May 2022 to April 2023 (10 months period). This rate varies from 0.0028% (Kota Kinabalu, Malaysia) to 0.1785% (Paris, France) with an overall rate of 0.038%.

As there is a lack of epidemiological studies on acute mesenteric ischaemia, this original study is more than welcome and we would like to congratulate the author for providing the first multicenter prospective study on the matter and one of the largest with 418 patients included. Even more, this study did not differ from the study protocol that the authors published in 2022 [2] where the primary objective—the identification of AMI incidence—was clearly stated.

We would also like to add some comments regarding the methodology applied. The reported high heterogeneity (*I*^2^ = 91%) of the overall incidence of AMI measured was intriguing. A heterogeneity of this importance is usually found in meta-analyses—for example the one by Tamme et al. [3] published in 2022 where they estimated the incidence of AMI in the general population based on 5 population-based studies (*I*^2^ = 100%)—but not in prospective trials. One of the reasons could be the high variation of incidence rates across centers suggesting actual difference between countries or continents on AMI occurrence. Since most published epidemiological studies regarding AMI originate from Northern Europe, we have no available data from Asia or South America and the actual incidence of AMI in non-Caucasian populations is unknown. And this is where, in our opinion, the problem lies as the AMESI study does not provide an actual true incidence of AMI.

By measuring the incidence of AMI among hospitalized patients, this study is inherently subject to a referral bias which occurs when a study mixes patients from non-tertiary and tertiary care centers (where patients from other centers are referred). The incidence of AMI among hospitalized patients from tertiary centers may be greater than in other centers, and this difference may be explained by the inclusion of referred patients. In the AMESI study, an information is given about patients that are referred in the supplemental Table 5 counting 114 patients included that did not originate from the inclusion site. They numbered 178 patients admitted from the emergency department, but did not provide information about the other 126 patients. This proportion of referred patients is therefore high representing at least 114/418 (27%) and at most 114/292 (39%) of the total cohort. This bias is perfectly illustrated by the center where the higher incidence has been found, Paris, with 77/78 (99%) of referred patients which is explained by the fact that this center has a specialized Intestinal Stroke Center unit.

The authors briefly discussed this interference of referred patients in the measure of outcome (which are stated as secondary objectives); however, referred patients were not excluded for the primary analysis yielding an overestimation of the incidence of AMI worldwide. In this setting, we believe actual true epidemiological difference across centers, countries or event continent cannot be extracted.

## Data Availability

Not applicable.

## Abbreviation

AMI: Acute mesenteric ischaemia
